# An Effective Package of Antioxidants for Avoiding Premature Failure in Polypropylene Random Copolymer Plastic Pipes under Hydrostatic Pressure and High Temperature

**DOI:** 10.3390/polym13162825

**Published:** 2021-08-22

**Authors:** Enrique Blázquez-Blázquez, Joaquín Lahoz, Ernesto Pérez, María L. Cerrada

**Affiliations:** 1 Instituto de Ciencia y Tecnología de Polímeros (ICTP-CSIC), Juan de la Cierva 3, 28006 Madrid, Spain; enrique.blazquez@csic.es (E.B.-B.); ernestop@ictp.csic.es (E.P.); 2 Centro de Ensayos, Innovación y Servicios, S.L. (CEIS). Cr. Villaviciosa de Odón a Móstoles (M-856) Km. 1.5, 28935 Móstoles, Spain; jlahoz@ceis.es

**Keywords:** PP-R pipes, monoclinic and orthorhombic polymorphs, new and aged pipes, fillers, antioxidants

## Abstract

Pipes of polypropylene random (PP-R) copolymers are the best choice for hot- and cold-water networks. Validation of a severe test, accomplishing the ISO 1167 standard, is mandatory to assess their service lifetime expectancy. This work evaluates the behavior shown by three commercial pipes, either the original ones (new pipes) or after being subjected to a hydrostatic pressure test at elevated temperature (aged pipes). Several features with relevance for the final performance have been examined: crystalline characteristics, phase transitions in crystalline regions, effect of high temperature and pressure on these transitions, and oxidation induction time. Moreover, the presence of inorganic fillers, and the content of different antioxidants together with their depletion, have also been analyzed. Films from the new pipes were also prepared for replication of the different environments in order to achieve a better and complete understanding of the phase transitions in the crystalline regions and of the consumption of antioxidants. Distinct environments surrounded the inner and outer parts of the pipes exposed to the failure aging test at 110 °C: hot water and warm dry air, respectively. These features play a key role in the loss of additives and in the subsequent initiation of degradation. Even if the crystalline characteristics are appropriate in the polymeric matrix, the success of a pipe lies in the homogeneous dispersion of components for avoiding damage at interfacial properties, and in a correct package of antioxidants used in its formulation.

## 1. Introduction

The market for plastic pipes has undergone an exponential increase in industrial applications over the past 50 years because of the many advantages exhibited by thermoplastic polymers, such as chemical resistance properties, low density (implying an important reduction in weight), negligible costs of external protection (painting, galvanizing, etc.) and high deformation capacity. In fact, the most commonly used piping material for potable water prior to 1970 was copper, but its high cost and the ease of installation of plastic pipes led to its replacement. Isotactic polypropylene (iPP or simply labeled as PP) is now the best choice in cold and warm potable water pipe networks. In addition to its lightweight nature and low cost, its attractiveness lies in its capability to be used at a relatively high temperature, unlike other thermoplastics used in piping, such as polyvinyl chloride (PVC) or high-density polyethylene (HDPE or simply named as PE).

The PP employed in pressure piping systems for high-temperature uses, such as in-house hot water supply and many other industrial piping applications, is a special PP grade, called PP-R. The term PP-R means, according to the definition found within industry standard ASTM F2389, polypropylene random copolymer, which is based on propylene and at least one comonomer, where the propylene is more than 50% of the composition. PP-R piping products are considered for continuous operation at a temperature of 82 °C, with pressure ratings dependent on their wall type. Ethylene or 1-butene are the common comonomers in PP-R and their incorporation leads to a decrease in crystallinity, rigidity, melting and glass transition temperatures in the resultant materials, compared with those properties exhibited by a PP homopolymer [1,2,3,4]. These characteristics provide a high elasticity and a great impact resistance to the PP-R pipes, which are very advantageous for their final use.

The good physical, chemical and mechanical characteristics exhibited by these PP-R pipes, together with the low cost of installation and maintenance, have been important aspects for their quick and outstanding expansion in the market. PP-R pipes also provide, among other factors, safety of potable water and long-term reliability; resistance to corrosion; chlorine and chloramine resistance; light weight and easy transportation; durability and toughness; availability in a wide range of sizes; and insulator nature showing low thermal conductivity. Furthermore, PP-R is a recyclable material, and PP-R pipes, whose shelf life is expected to be at least 50 years, could contribute to the circular economy at the end of their use. Both aspects, recycling and durability, are important in the actual context for the preservation of the environment.

The aforementioned long-term service requires accurate failure prediction tests. For that, both experimental and theoretical experiments have been implemented. The former involve international standard methods to be applied for estimation of lifetime in plastic pipe systems, such as the standards ISO 19893 or ISO 1167 [5,6]. They specify a protocol for determination of the pipe’s resistance to internal hydrostatic pressure at a given temperature in thermoplastic materials for the transport of fluids. Those pipes tested in accordance with the standard and showing no bursting or leakage are supposed to be capable of lasting over 50 years in service, since they will resist the hydrostatic stress induced by the internal hydrostatic pressure. Regarding theoretical tests, these make use of statistical models to calculate the optimal replacement time for pipes [7,8,9].

Knowledge of long-term failure behavior is essential and mandatory for making changes and fits in the PP-R pipe fabrication system, as well as in the selection of manufacturing materials to ensure a correct performance. The combined effects of pressure and temperature, together with the chlorine treatment of potable water, could lead to microstructural, morphological and structural variations of PP-R crystalline characteristics, as well as consumption of additives [10,11,12] in these pipes. All of these changes can affect parameters as important as the degree of crystallinity or crystal structure, which will negatively alter the overall mechanical response of the pipe. An additional deterioration could occur in pipes formulated with a deficient package of antioxidants, since a premature consumption of these additives will boost the PP-R susceptibility to oxidation, leading to initiation of an embrittlement phenomenon. Sometimes, improvements in the final performance do not require important technical alterations but the scientific comprehension of basic aspects. Thus, novelty deals with examining the evidence related to microstructural, morphological and structural changes, together with the antioxidant package incorporated and antioxidant loss as an attempt to achieve an explanation for the problem.

The aim of this research is, therefore, to understand the reasons behind the different behavior exhibited by three distinct commercial PP-R pipes, which have been subjected to an accelerated aging test in accordance with the ISO 1167 standard, in order to improve future formulations for ensuring the long-term pipe lifetime (achieving the ISO 1167 protocol). For this purpose, three original pipes have been examined, together with the same three after exposure to the accelerated pressure-temperature aging treatment. Furthermore, it has been taken into account that the accelerated aging could affect differently the interior and exterior parts of the pipes, since the former has been in direct contact with hot water at a pressure of 6 bar and the outer section is under the action of warm dry air at 110 °C, which is the temperature of the convection oven where thermal treatment was performed. Therefore, crystalline characteristics, phase transitions, morphological aspects, oxidation induction time and analysis of additives have been thoroughly examined in both inner and outer pieces of aged pipes, and compared with those obtained from the original new ones. In order to gain information about some features of phase transitions and about the early stages of degradation promoted by consumption of additives, films from the original pipes were also prepared and aged, attempting to reproduce some of the conditions (temperature and surroundings) used in the internal hydrostatic pressure test.

## 2. Materials and Methods

Three commercial Ziegler-Natta PP-R materials, designated as PP-R_061, PP-R_490 and PP-R_826, were analyzed. Melt flow indexes listed in their technical data sheets were 0.24 g/10 min, 0.3–0.4 g/10 min and 0.3–0.4 g/10 min, respectively, all of them determined at 230 °C/2.16 kg. Moreover, glass fiber in a weight content of 6% was reported for the PP-R_061 material.

The corresponding commercial pipes obtained from those PP-R materials were supplied by CEIS (Test, Innovation and Services Center). They were named as P061, P490 and P826. These original pipes will be designated as new. Figure S1 of the Supporting Information shows a transversal cut for each of the new pipes. The gray layer located in the middle of the P061 pipe is the portion containing glass fiber.

Failure aging tests for evaluation of long-term resistance of those pipes to an internal hydrostatic pressure of 6 bar at 110 °C was performed by personnel of CEIS. In accordance with the ISO 1167 standard, the pipes must resist at that pressure and temperature for times longer than 8760 h. The tested pipes had the same geometrical features, with an external diameter of 20 mm and a thickness of 3 mm.

Films from the original new pipes were obtained by compression molding in a Collin press between hot plates at a temperature of 180 °C and a pressure of 25 MPa for 3 min. Two different thermal treatments from the melt were applied. The first one consisted of a slow cooling to room temperature at the inherent cooling rate of the press after the power was switched off (the cooling rate was around 1.5 °C/min). Pressure was maintained constant at 25 MPa along this treatment. It is designated as S. The second protocol involved a relatively fast cooling (around 100 °C/min) from the melt to room temperature between plates of the press refrigerated with cold water. This film is labeled as Q.

In order to reproduce the aging protocol (for the evaluation of some aspects in the phase transitions and the initial stages of degradation because of consumption of additives), the obtained films were placed in an oven at 110 °C for 18 days. Some portions were directly exposed to warm dry air (emulating the outer part of the pipe, and named as F061Q18A, F061S18A, F490Q18A, F490S18A F826Q18A and F826S18A) or immersed in water at 110 °C within closed glass vessels (in order to reproduce the inner section and designated as F061Q18W, F061S18W, F490Q18W, F490S18W F826Q18W and F826S18W). All of the samples used in this investigation are summarized in Table S1 of Supporting Information.

Wide-angle X-ray diffraction (WAXD) experiments were carried out with synchrotron radiation in a beamline BL11-NCD at ALBA (Cerdanyola del Valles, Barcelona, Spain) at a fixed wavelength of 0.1 nm. A Rayonix detector was used at a distance of about 19 cm from the sample and a tilt angle of around 30°. A Linkam Unit, connected to a cooling system of liquid nitrogen, was employed for the temperature control. The calibration of spacings was obtained by means of silver behenate and Cr_2_O_3_ standards. The initial 2D X-ray images were converted into 1D diffractograms, as a function of the inverse scattering vector, *s* = 1/*d* = 2 sin *θ*/λ. Samples of around 5 × 5 × 0.2 mm were used in the synchrotron analysis.

The phase transitions were analyzed by DSC in small pieces (about 5 mg) of new and aged PP-R pipes. Analyses were also conducted in the samples reproduced in a laboratory convection oven, as aforementioned. Experiments were performed in a TA Instruments Q100 calorimeter connected to a cooling system. A temperature interval from 25 to 190 °C was studied at a scanning rate of 20 °C/min under a nitrogen purge. The first melting processes were evaluated in detail. For the determination of the crystallinity, a value of 160 J/g [13,14] was used as the enthalpy of fusion of a perfectly crystalline material. The values of T_m_ were obtained from the maximum of the endothermic peak and the enthalpy of melting (ΔH_m_) was estimated from the integral of the area below the endothermic events.

Micrographs were acquired at room temperature in a PHILIPS XL30 ESEM environmental scanning electron microscope working at 25 kV, using a secondary electron (SE) detector and a magnification of ×6500 for the morphology studies. The samples were cryofractured prior to observation.

Optical micrographs were obtained at room temperature in a Leica DMS1000 stereomicroscope with an objective achromatic. Images were taken at a magnification of ×4.0 for morphological analysis.

Bulk portions of the different pipes were placed in a P-Selecta oven at 650 °C to perform their calcination in order to analyze the inorganic components. The resulting powder was analyzed using a Perkin–Elmer Spectrum-One FTIR equipped with a total attenuated reflectance device (ATR), with 16 scans and resolution 4 cm^−1^. Furthermore, the elemental composition of the inorganic powder was determined by energy-dispersive X-ray (EDX) spectroscopy combined with the PHILIPS XL30 ESEM environmental scanning electron microscope mentioned above.

Oxidation induction time (OIT) was analyzed in a Mettler Toledo DSC822e differential scanning calorimeter (DSC). Samples of about 5 mg were deposited in open aluminum pans. Samples were heated to the test temperature (210 °C) at a rate of 20 °C/min under a nitrogen atmosphere and maintained at that temperature for 3 min before beginning oxidation. Then, purge gas was commuted to oxygen and the heat flow was recorded versus time. The OIT value was determined from the time of the onset of the oxidation process. Tests were duplicated five times per pipe analyzed, taking pieces separately located in different similar sections of the pipes.

A section of around 1 g for each pipe, either new or aged, was cut in order to analyze the additive content. Then, additives were extracted in a Soxhlet with dichloromethane for 8 h. The extract was concentrated in a rotary evaporator. The obtained residue was transferred to a chromatographic vial and dried with a nitrogen flow. Then, residue was re-dissolved in a specific volume of dichloromethane. The analytical determination was carried out using a Hewlett Packard 6890 GC gas chromatograph equipped with an Agilent Technologies mass spectrometry detector model 5973 (GC-MS technique). The separation of the compounds was performed on a DB5-HT capillary column (15 m length, 250 µm internal diameter and 0.1 µm film thickness). The carrier gas was helium with a flow rate of 1 mL/min. A volume of 1 µL of the obtained extract was injected in split mode with a split ratio of 20:1, at 270 °C. The electronic impact (70 eV) was the selected type of ionization for the mass spectrometer. The chosen chromatographic method lasted 37.5 min. The program started at 80 °C, temperature was increased at a constant rate of 8 °C/min up to 340 °C, and was maintained for 5 min [15].

## 3. Results and Discussion

### Aging Test, Crystalline Characteristics and Melting Processes

Time before failure during the aging test for predictions of the long-term resistance in the different pipes is detailed in Table 1. Different behaviors are shown for pipes under a constant hydrostatic internal pressure of 6 bar at a temperature of 110 °C. The P490 pipe failed after 6256 h, the P061 pipe after 8338 h and the P826 pipe was the only one that lasted longer than the time established by the ISO 1167 standard, i.e., >8760 h. Test duration was dependent on the tension generated through the pipe by these two parameters: the internal pressure and the temperature. Formation of creep or burst was detected through an internal pressure testing system.

Several pieces (IN and OUT) from either new or aged pipes were evaluated in order to understand the reasons why the three commercial pipes showed these distinct responses to the aging internal pressure test at high temperature. In addition, films prepared with two thermal histories (S and Q) and exposed to two different environments (air and water) were also analyzed. The list of all of them, as well as their brief description, is reported in Table S1 of Supporting Information.

Firstly, the different variables (temperature, pressure, warm dry air and hot water) taking part in the aging test can exert an important effect on the crystalline structure of the pipes. For that, the characteristics related to the crystalline lattices developed in the inner and outer sides for the different new pipes were evaluated by X-ray experiments using synchrotron radiation. The iPP-based materials, in general, can develop several polymorphs by changing their microstructural features, the crystallization conditions and other factors like incorporation of specific nucleants [16,17,18,19]. Thus, three different polymorphic modifications, α, β and γ, have been reported together with a phase of intermediate or mesomorphic order obtained by application of a fast quenching [16,18,19,20,21,22]. All of these lattices share a three-fold conformation. In addition to these four modifications, a trigonal form was described more recently for isotactic copolymers of propylene with high contents of 1-hexene [23,24,25] or 1-pentene [26,27], in terpolymers of propylene with 1-pentene and 1-hexene [28,29] as comonomers, and in propylene terpolymers with 1-pentene and 1-heptene [30]; these copolymers and terpolymers were synthesized by using metallocene catalysts.

**Figure 1:**
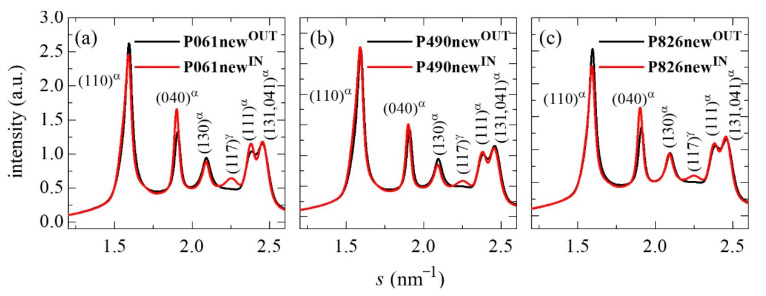
1D WAXD profiles for the initial outer (new^OUT^) and inner (new^IN^) parts of the different pipes analyzed: (**a**) P061; (**b**) P490; and (**c**) P826.

Figure 1 shows the 1D diffractograms at room temperature from the outer and inner parts of the several new pipes. It is noticeable that the patterns for the outer parts in all of the distinct pipes analyzed do not exhibit the reflection located at around 2.25 nm^−1^, which is clearly seen in their corresponding inner counterparts. These WAXD profiles in the outer sides display the characteristic main reflections for the monoclinic α lattice exhibited by iPP: the (110), (040), (130), (111) and (041, 131) reflections. The additional diffraction observed in the inner sections is ascribed to the presence of an amount of the orthorhombic γ polymorph, and corresponds to its (117) distinctive diffraction. The content in γ form can be deduced from the relative intensities of the (130) monoclinic α reflection [31] with respect to that from the (117) reflection stemming from the orthorhombic cell [32]. Values obtained for the relative percentage of γ polymorph are 26, 21 and 17 in the P061new^IN^, P490new^IN^ and P826new^IN^, respectively, while these are 74, 79 and 83 for the α modification in the P061new^IN^, P490new^IN^ and P826new^IN^, respectively.

The orthorhombic form is only developed in the inner part of pipes. The most common and stable crystalline structure in iPP is the monoclinic α polymorph. The orthorhombic γ lattice was found in low molecular weight iPP [33,34,35] and in random copolymers of propylene and α-olefins, like the current ones, favored when increasing the proportion of comonomer incorporated [36]. Moreover, the γ modification is also specially boosted in iPP synthesized by metallocene catalysts due to the presence of errors homogeneously distributed among polymeric chains [37]. The γ form in polypropylene-based materials is more easily developed when crystallization is performed at relatively low rates [3,31,32]. The standard processing used in industry for the manufacture of pipes implies that the outer side is cooled faster than the inner parts, since the heat dissipation is slower in the inner parts than in the outer parts. This means that interior macromolecules undergo a lower cooling rate than those located in the exterior of the pipes. Consequently, they will crystallize at a higher temperature than those in the exterior, as can be seen from the phase diagram described in the literature [38]. This fact makes it possible that polymeric chains are able to develop in the pipe interior either monoclinic (in the majority, as aforementioned) or orthorhombic (in a minor amount) crystallites, while those in the external sections only crystallize in the monoclinic form. This difference in the cooling rates at the inner and outer parts of pipes seems to lead also to a slightly higher crystallinity in the former, as deduced from the intensity of the reflections in the profiles represented in Figure 1 and from the values estimated from the profiles: 0.55 and 0.52 for P061new^IN^ and P061new^OUT^; 0.55 and 0.53 for P490new^IN^ and P490new^OUT^; and 0.53 and 0.50 for P826new^IN^ and P826new^OUT^.

**Figure 2:**
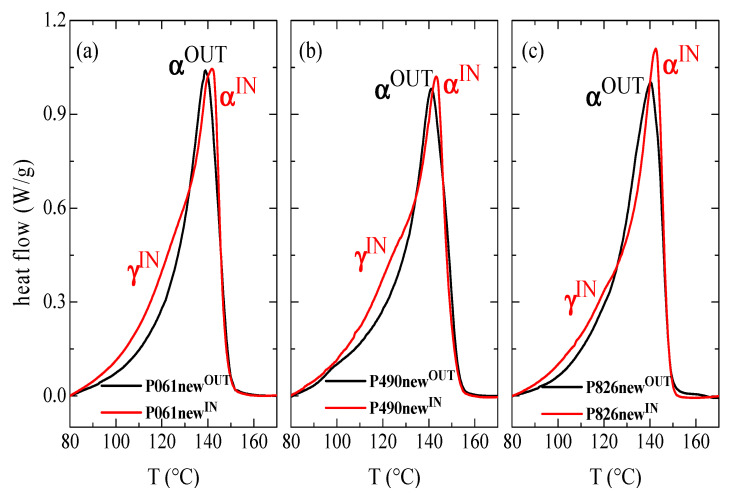
First DSC melting curves of the inner and the outer parts in the different new pipes: (**a**) P061; (**b**) P490; and (**c**) P826.

In order to obtain deeper information of the crystalline characteristics for the different new pipes, DSC experiments were carried out. Figure 2 shows the first heating curves for the interior and exterior parts in these new pipes, and the magnitudes derived from them (enthalpy, crystallinity and temperature of the melting processes) are listed in Table 2. Crystallization temperature obtained from the cooling DSC run at 20 °C/min has also been added for all of them.

Several differences are found when outer and inner parts are compared for a given pipe. The most evident is that the maximum of the curves, from where the T_m_ value is attained, is shifted to slightly higher temperatures in those curves corresponding to the interior parts of pipes, i.e., in P061new^IN^, P490new^IN^ and P826new^IN^. Accordingly, the T_m_ is around 2.5 °C lower in the different samples taken from the pipe exterior, independently of the pipe considered, i.e., in P061new^OUT^, P490new^OUT^ and P826new^OUT^. This endothermic process corresponds to the melting of the monoclinic α crystallites. However, there are not significant variations between the different pipes analyzed.

Moreover, a small shoulder in the low-temperature region is also observed in the endotherm of the inner parts, i.e., in P061new^IN^, P490new^IN^ and P826new^IN^. This is more clearly distinguishable when comparison between the endotherms of the outer and inner parts is performed, the curve being almost unimodal in the former. This shoulder is ascribed to the melting process of the minor amount of orthorhombic γ crystals (as deduced from the synchrotron experiments at variable temperature), which coexist with the monoclinic ones in these inner samples for the different new pipes. The γ crystallites are smaller and thinner than those monoclinic ones and, therefore, melt at lower temperatures [39,40,41]. These γ crystallites are formed at higher temperatures although they are less stable than the monoclinic ones, and their T_m_ is shifted to lower temperatures [39]. In fact, the shoulder appears in the temperature interval ranging from 100 to 130 °C.

**Figure 3:**
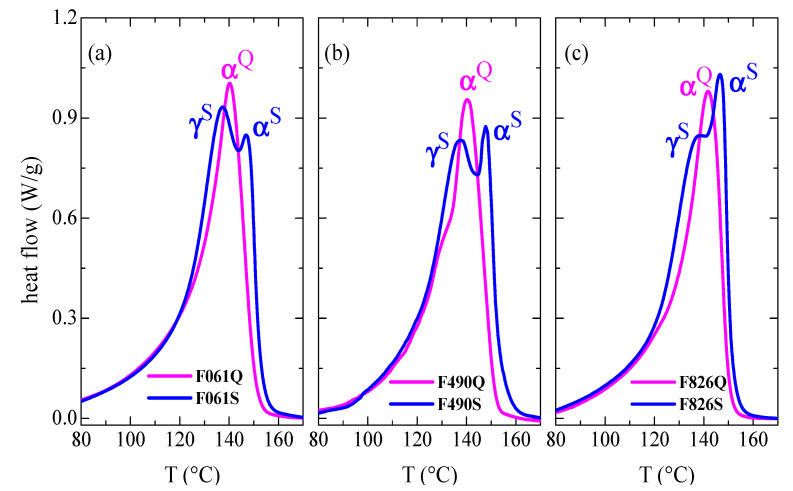
First DSC melting curves obtained from quenched and slowly cooled (Q and S) films prepared from the different new pipes: (**a**) P061; (**b**) P490; and (**c**) P826.

A greater splitting of this main melting peak, due to the coexistence of the γ and α crystallites, is observed if content in the orthorhombic lattice is the majority [39,40,41], as seen in Figure 3. This figure displays the DSC curves for the Q and S films processed from the different new pipes to reproduce the internal and external sides (see Experimental section) to a larger extent. Accordingly, the S thermal treatment applied during processing implies a cooling that is much slower than that imposed during pipe manufacture. Thus, a considerable increase in intensity for the melting process related to γ crystals, which is the peak appearing at the lowest temperature in the S films, is seen when a slow crystallization has been carried out during cooling. Formation of the orthorhombic lattice is significantly boosted at low rates, becoming the major crystalline lattice developed in the films [39,40,41]. The γ crystals are generated in a greater amount in the F061S film, since the peak located at the lowest temperature exhibits an intensity higher than the one corresponding to the α crystallites found at higher temperature. This content in γ polymorphs slightly decreases in F490S and now both peaks display a similar intensity. The diminishment is even more significant in F826S films and the melting of the γ form is just observed as a shoulder overlapped to the α endothermic peak. However, DSC curves for the initial Q samples are rather analogous to those observed in the outer parts of the different new pipes (see Figure 2). The monoclinic entities are in majority in these quenched films. Figure 3 also displays perfection of the monoclinic α crystallites developed under slow cooling (S films). Their larger size, compared with the α crystals formed during the quenching applied in the Q films, leads to their superior melting temperatures, T_m_^αS^. During the Q treatment, crystallization took place at conditions far from equilibrium, leading to thinner monoclinic crystallites. This is also the reason why T_m_^αIN^ in the inner sections is shifted to slightly higher temperatures compared with the T_m_^αOUT^ observed in the outer parts.

In addition, a third feature that can be derived from the comparison of DSC curves for the inner and outer parts of the new pipes, represented in Figure 2, is related to the melting enthalpy (degree of crystallinity developed). Values are sensibly higher in P061new^IN^ and P490new^IN^ with respect to those found in their corresponding P061new^OUT^ and P490new^OUT^ counterparts. Crystallinity is, however, more similar for the two pieces of P826 pipe, its value in P826new^IN^ being only slightly higher than in P826new^OUT^. These results, reported in Table 2, corroborate those deduced from X-ray diffraction.

All of these crystalline aspects indicate that the industrial manufacturing process leads to a structural heterogeneity along the complete section of the original pipes. This heterogeneity is not only related to the existence of crystallites with different sizes, depending on being located in the inner, bulk or outer parts of pipes (which is associated with the particular rate of cooling that they have undergone during processing), but also with the type of polymorph developed (monoclinic or orthorhombic) and with its unique presence or the coexistence of both crystalline lattices.

**Figure 4:**
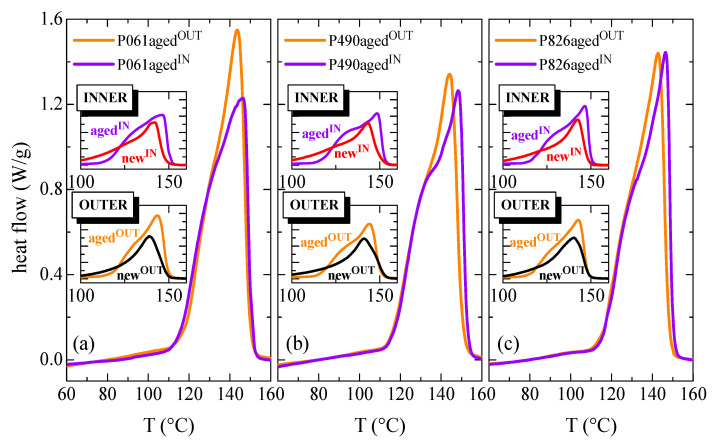
First DSC melting curves for the inner and the outer parts in the different aged pipes: (**a**) P061; (**b**) P490; and (**c**) P826. The insets show for both the inner and outer sections the comparison of results between aged and new pipes.

For the different aged pipes, the effect on crystalline transitions of the internal hydrostatic pressure test, performed at 110 °C, is deduced from Figure 4. Consequently, the DSC curves are shown for their outer and inner aged sections, together with the comparison with the corresponding curves for the exterior and interior parts of the new pipes (represented in the different insets of Figure 4).

As discussed previously for the new pipes, certain differences are found in the aged pipes between the outer and inner parts for a specific pipe. Again, the T_m_^αIN^ value exhibited by the inner section is higher than T_m_^αOUT^ observed in the corresponding exterior counterpart, because macrochains have crystallized under slightly more favorable conditions. The highest variation is found in the P490 pipe, while the smallest change is observed for the P061 pipe, as listed in Table 2.

It can be also deduced by comparing between aged and new pipes that the T_m_^α^ values are in the aged pipes higher than those observed in the new ones. Accordingly, aged crystallites appear to be thicker than the existing crystals in the new pipe. Maintenance at 110 °C during the internal hydrostatic pressure test has allowed perfection of all of the crystallites existing within the pipes, together with the development of a larger amount of crystalline fraction. Consequently, the degree of crystallinity has also been significantly enlarged in the aged pipes, either in the inner or outer parts, compared with that exhibited in the corresponding ones from the new pipes. This is evident from the area involved in the endotherms represented in the different insets of Figure 4.

Regarding the crystalline lattices existing in the aged pipes, it is expected, as in the new pipes (see Figure 1), that there will be coexistence of monoclinic and orthorhombic crystallites in the inner section, while only monoclinic crystals will exist in the outer part, since crystal→crystal transitions from orthorhombic to monoclinic lattice or vice versa have never been described by the effect of the annealing (in the present case occurring during the course of the internal hydrostatic pressure test at a high temperature). Nevertheless, the appearance of a shoulder is now also observed in the outer parts of aged pipes (see comparison between the exterior sections for a specific pipe in insets of Figure 4) while in the new ones that shoulder is only seen in the inner part, being ascribed to the melting of the orthorhombic crystals. Although only monoclinic crystals are developed in the outer parts, performance of the internal hydrostatic pressure test at 110 °C has provoked some important changes in the crystalline regions. In the outer parts, where monoclinic entities were exclusively formed during manufacturing, the stay at that high temperature for long time has allowed the melting of the smaller and thinner monoclinic crystals, whose T_m_^α^ was lower than 110 °C, leading to their further recrystallization as thicker and more perfect monoclinic crystallites. This phenomenon has been commonly labeled as the annealing effect [42]. This crystal population is the one that contributes to the melting process observed at around 120 °C previous to the main melting peak [43,44]. These melting-recrystallization processes are not specific to propylene-based materials, also appearing in other semicrystalline polymers [45,46,47,48,49].

Concerning the inner sections of pipes, where coexistence of monoclinic and orthorhombic crystallites takes place, several effects are overlapped in that shoulder. Maintenance at 110 °C during the pressure test allows perfection of either the thinner monoclinic α entities or the orthorhombic γ ones through consecutive melting-recrystallization processes. These thicker monoclinic crystals generated from the initially thinner ones will melt at temperatures slightly higher than the temperature applied during the pressure test. In addition, the melting of the more perfect orthorhombic crystallites, whose size has been also enlarged during the test, will also occur. A perfection of these γ crystals in these inner aged pipes compared with those initially formed in the inner new pipes is easily deduced for the shift of the shoulder to a higher temperature, as clearly seen from the insets, denoted as INNER, of Figure 4. The increase in intensity in the shoulder is associated with the larger amount of these γ crystals and also with the contribution to enthalpy of the melting of those thinner monoclinic crystallites that were molten and recrystallized during the pressure test. The important aspect is that, independently of the polymorph involved (and of being IN or OUT side), the melting endotherm in the aged samples is significantly narrower than in the original ones.

**Figure 5:**
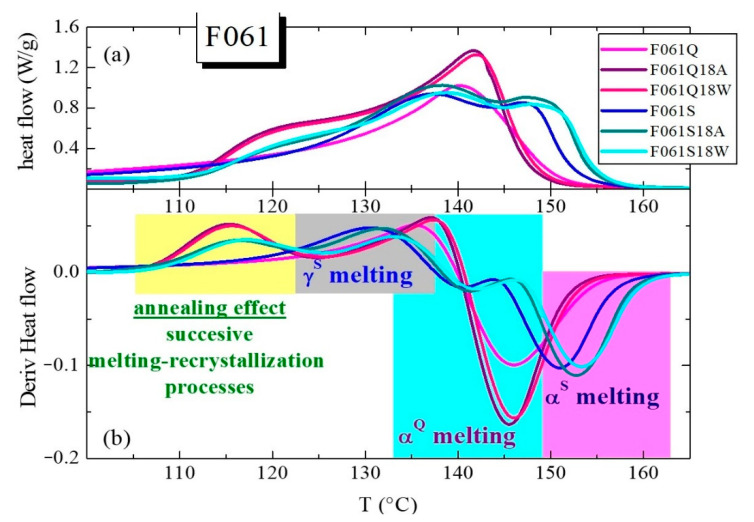
(**a**) DSC curves obtained for P061-based materials: Q and S (F061Q and F061S) films and their emulated thermal treatments in warm dry air (F061Q18A and F061S18A) or immersed in water (F061Q18W and F061S18W). (**b**) Derivatives corresponding to the different DSC curves.

Derivatives of DSC curves can constitute a very useful tool to verify the aforementioned annealing effect and the other melting processes. In the upper plot of Figure 5, DSC curves are represented, as an example, for the F061Q and F061S films, together with those prepared from them to reproduce the inner and outer parts of the pipes, designated as F061Q18A, F061Q18W, F061S18A and F061S18W (see Table S1 in Supporting Information for description). These specimens have been chosen because the content in γ crystals is the highest in the films derived from the S thermal treatment and, then, the usefulness of DSC derivatives (depicted in the lower plot of Figure 5) is more evident.

The advantage of these derivative curves is that they display different peaks corresponding to the maximum variations in the heat flow, so that they offer an easy alternative for discerning the different processes involved. Thus, the annealing effect is clearly noticeable in all the specimens that have undergone the thermal treatment at 110 °C. Evidently, it is not observed in the F061Q and F061S films because they have not been subjected to that thermal treatment at 110 °C. Derivatives are also very illustrative for the S samples, since the presence of two clear melting processes is discernible beyond the annealing process observed in the treated specimens. These two endothermic events correspond to the coexistence of the two different γ and α polymorphs, as mentioned above. In the Q films, a simpler behavior is seen. The F061Q film only shows the process ascribed to the melting of its monoclinic crystallites, while the F061Q18A and F061Q18W specimens display the annealing process at low temperatures along with the main melting of those thicker α crystals.

This study of the existing crystalline polymorphs and their phase transitions has not shown important variations between the pipes. It indicates that there are not significant microstructural differences (molecular weight, comonomer content) between the distinct PP-R materials used for the manufacturing of these commercial pipes. Some changes have been observed in all of them between the external and internal parts for a given pipe derived from the fabrication process: the presence of a unique crystal lattice or the coexistence of two polymorphs. Nevertheless, these variations do not justify the dissimilar behavior exhibited by the time before failure during the normalized aging test in the different PP-R pipes, mainly in the P490. Thus, the next aspect to be checked, which could have some influence, is the analysis of the existence of any filler within the different pipes.

**Figure 6:**
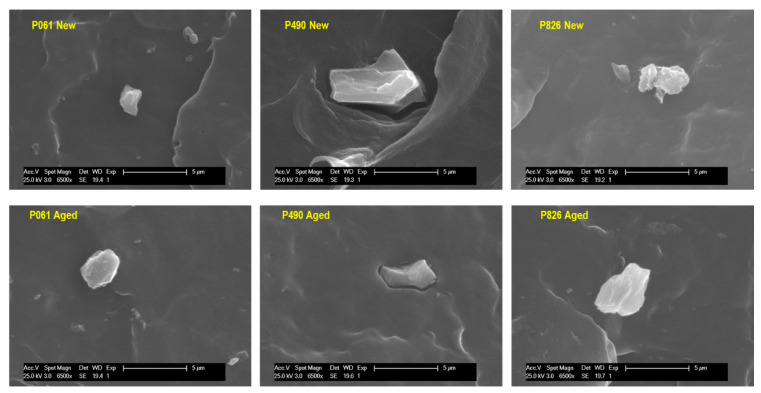
SEM micrographs obtained from cryofractured surfaces for the indicated pipes.

The presence of inorganic components could contribute to increased heterogeneity. Such components can also boost interactions, in the manufacturing process or in the aging test, of the existing oxidizing agents with the pipe material, leading to early degradation [15,50]. This phenomenon can be overstated by the presence of inorganic fillers with a tendency to form aggregates, which would avoid a suitable interfacial adhesion to the polymeric matrix [51]. Accordingly, an analysis of fillers was performed by SEM in cryofractured samples taken from the new and aged pipes. Figure 6 shows the existence of inorganic particles, with different shapes and sizes, in the distinct pipes.

In order to identify the nature of the additives, calcination of pipes was carried out and, afterward, the powder achieved was further analyzed by FTIR (see Figure S2 in the Supporting Information) and SEM-EDX (see data in Table S2 of Supporting Information). Results indicate that the pipe P826 only contains TiO_2_; the P061 pipe incorporates TiO_2_ and glass fiber; and the P490 pipe is filled with TiO_2_, CaCO_3_ and small amounts of other metals such as Fe or Zn. Addition of pigments during extrusion is a common practice in the manufacturing process [52,53]. The 0.9 wt.% in CaCO_3_ found in the P490 pipe could be related to an inorganic pigment added as a filler [54]. This type of inorganic incorporation makes the use of coupling agents convenient in PP-based materials [55,56,57] to improve the interfacial properties in the resultant polypropylene/CaCO_3_-based composites. If coupling agents are not employed, the inorganic particles can act as stress concentration sites, leading to formation of voids at the particle boundaries [58]. Furthermore, the action of temperature and pressure during the aging test, together with the effect of the salts that water can contain, could favor the solubility of CaCO_3_ [59] and provoke formation of a greater heterogeneity on the surface of the pipe.

Figure 6 clearly shows the existence of voids in the P490 pipe [58], where the CaCO_3_ particles exhibit a more polyhedral form. These features are in agreement with those found previously in polypropylene composites [60]. The holes observed near the particles in the P490 pipe could constitute points of access for greater contact between the oxidizing agents from the surroundings, boosting formation of initial crazes and their growth to cracks.

**Figure 7:**
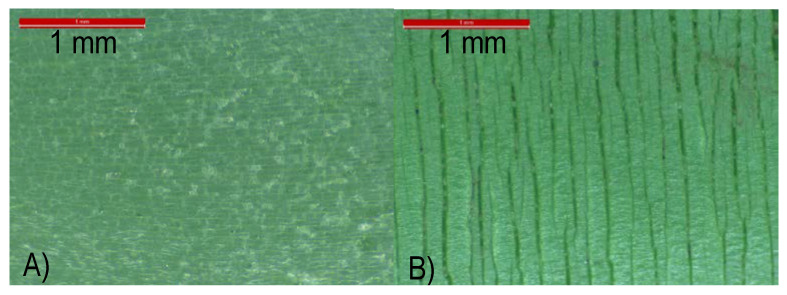
Optical micrographs for pipe P061 after aging test to internal hydrostatic pressure (P061aged): (**A**) inner part in contact with pressure and hot water; and (**B**) outer section in contact with warm dry air.

Another important difference between the several pipes studied is the presence of a glass fiber layer inside the P061 pipe (see Figure S1 of Supporting Information). Addition of these fibers contributes to reducing the extension capability of the pipes and to increasing the deflection temperature of polymeric materials [61,62,63,64]. Glass fiber could hinder or slow down propagation of the cracks that are developed during the chemical degradation of the material, which is mainly undergone during the aging test. Figure 7 shows the great difference existing between surfaces found in the inner and outer sides of the aged pipe P061. Small voids are seen in the interior side while much longer cracks are noticeable in the outer counterpart.

Evaluation of the fillers incorporated shows, then, significant differences between the pipes. Although all of them contain TiO_2_, the P061 pipe also incorporates glass fiber, whereas the P490 pipe is additionally filled with CaCO_3_. Presence of these inorganic components has not considerably affected the phase transitions previously evaluated because they are in low amounts in the distinct pipes. Nevertheless, their presence seems to contribute to increased heterogeneity of the final material and even formation of voids.

A second kind of additive used in polymer formulation, usually in very small proportions, comprises antioxidants, which are essential for optimization of ultimate properties and for extending the useful life of the polymer.

Knowledge of consumption of antioxidants and the oxidative features shown by the different pipes is therefore of great importance for understanding the results of failure aging tests for evaluation of their long-term resistance, as aforementioned in the Introduction. The thermo-oxidative characteristics of these PP-R pipes were assessed by the oxidation induction time (OIT) at a temperature of 210 °C. This method has been widely employed to evaluate the effectiveness of antioxidants in a given polymer when exposed to an oxidative atmosphere [65,66,67]. Primary and secondary antioxidants are commonly used in polymers to improve their performance during manufacture, processing and their service life [68,69,70,71]. Therefore, and previously to test the pipes, some of these common antioxidants were selected for assessment of their thermal behavior under identical OIT conditions to the ones used in the different PP-R pipes. Figure S3 of Supporting Information shows the curves obtained for Irganox 1010, Irganox 1076 and Irganox 1330 (primary antioxidants) and Irgafos 168 (secondary antioxidant). An important change is observed in the heat flow curve when an oxidation process occurs. As can be seen, an almost plateau stage is noticeable at oxidation times longer than 10–15 min in the primary antioxidants. This behavior indicates the good resistance of these compounds to thermal oxidation [11].

A different trend is observed in Irgafos 168, which is a secondary antioxidant. An important change in slope is noticeable in the curve just after the initial inert gas of the experiment is switched to oxygen. This feature has been ascribed to a fast oxidation of this phosphite-based stabilizer into its corresponding phosphate form when Irgafos 168 is under an oxygen environment [72]. There are several studies [73,74] that describe the high stability of phosphates, making them the commonly named non-intentionally added substances (NIAS) in food packaging [75,76]. Once the phosphite form is transformed into the phosphate compound, the shape of the heat flow curve is similar to that observed for the rest of the primary stabilizers evaluated, because of its high stability.

**Figure 8:**
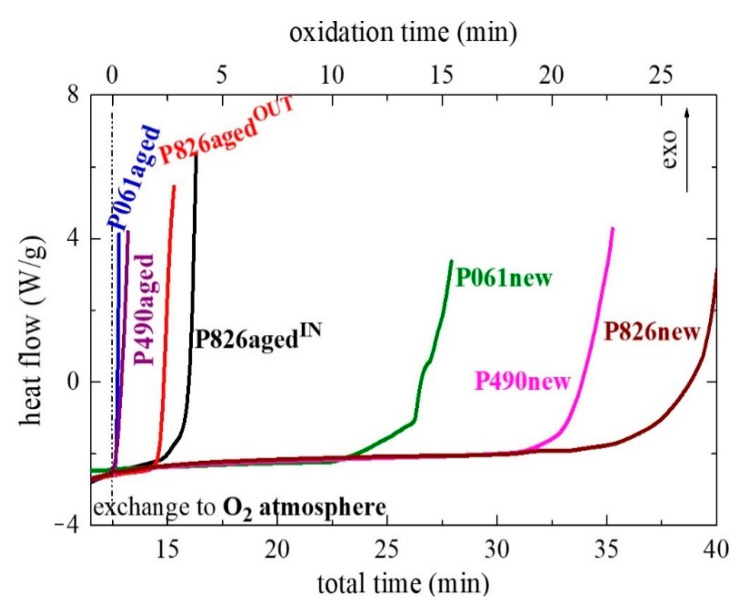
Thermo-oxidative response at a temperature of 210 °C for the different pipes. The oxidation stage begins at 12.5 min of total time.

Once these different common antioxidants were analyzed, the study was performed for the several new and aged pipes. A correct understanding of the results deduced from this technique has to take into account that the inner and outer pieces of the pipes are expected to undergo different rates of additive consumption because of the distinct aging environments to which each of them was subjected. The former is under the action of pressure, water and temperature, while the last is under the effect of dry air and temperature. Figure 8 shows the results for all of them. Significant variations are found between the several pipes analyzed. The original pipe P826, P826new, displays the best OIT response and, accordingly, the best thermal stability, with a value of 25 ± 0.5 min. Pipe P490new shows an intermediate result and its OIT is 20.5 ± 0.5 min, whereas pipe P061new exhibits the lowest value with 12.5 ± 0.5 min.

Comparison of these results with those listed in Table 1 for the internal pressure aging tests seems to indicate a certain disagreement. The pipe P826new displays the best OIT value and it is the one that satisfactorily passed the aging experiment, remaining intact for 8760 h. The OIT response for pipe P490new could point out an intermediate resistance to the internal pressure test. Nevertheless, it is the pipe that lasted the shortest time, only 6256 h. Finally, the pipe P061new exhibits the worst OIT value but its failure takes place after 8338 h in the internal pressure test. Is there any discrepancy in this behavior? Before answering this question, let us analyze the OIT response found in the aged pipes.

The two aged P826aged^IN^ or P826aged^OUT^ pipes, i.e., the pieces taken from the inner or outer part, respectively, are again the ones showing the highest OIT values. The P061aged and P490aged pipes show, however, a sharp exothermic process just immediately after the exchange from inert gas to oxygen. A differentiation is not made in Figure 8 between their respective inner or outer parts in these two P061aged and P490aged pipes because the two sides of them, i.e., P061aged^IN^ or P061aged^OUT^ and P490aged^IN^ or P490aged^OUT^, exhibit practically identical results. Accordingly, superposition of curves was complete for pipes P061aged^IN^ and P061aged^OUT^ and the same behavior was also found in the case of P490aged^IN^ and P490aged^OUT^. The important conclusion from Figure 8 is that the two aged P061aged and P490aged pipes have undergone a significant reduction in their OIT values, independently of taking the sample from their inner or outer parts. Moreover, OIT dependence is the same in the aged pipes to that found in the new ones, i.e., the OIT value decreases in the order P826 > P490 > P061.

Regarding the results in the P826aged^IN^ and P826aged^OUT^ pipes presented in Figure 8, they seem to indicate that the thermo-oxidative response in the former is better than the one exhibited by P826aged^OUT^. The action of pressure, water and temperature seems to be less harmful than the effects of dry air and temperature, although differences in their OIT values are not too large.

A complete evaluation of the type and amounts of antioxidants, as well as their loss, is therefore mandatory to understand the OIT results, since they are strongly dependent on the global content in primary and secondary antioxidants, as aforementioned. Reduction of their amount in the pipes can be also associated with the beginning of their deterioration, taking into account the aggressive conditions (in terms of time and temperature) used along the aging test (and also during the actual lifetime of the pipe).

**Figure 9:**
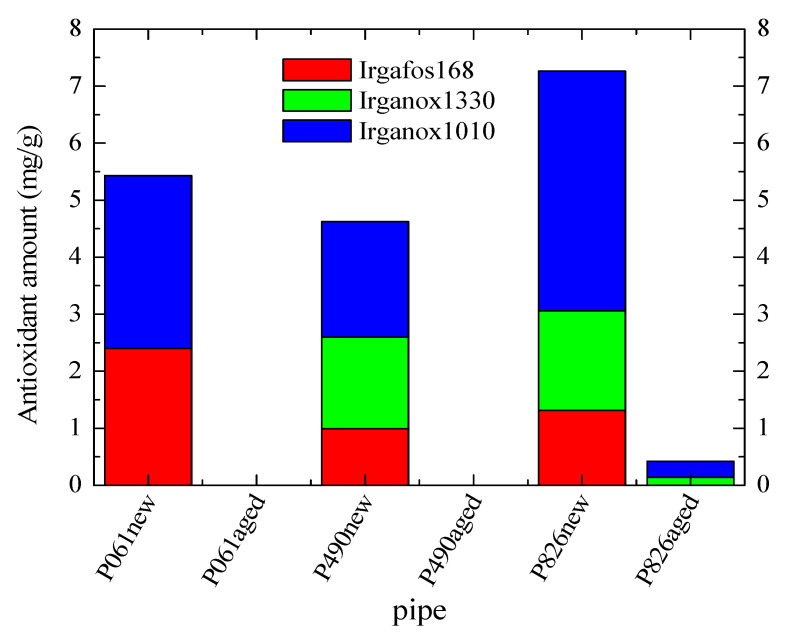
Antioxidant composition in the different new and aged pipes. The content of antioxidants in pipes P490aged and P061aged was found to be zero.

The nature and content of additives was determined by GC-MS. The results, shown in Figure 9, indicate that antioxidants (Irganox 1010, Irganox 1330 and Irgafos 168) are the only additives present in the original new pipes. The three of them are found in the pipes P826new and P490new, although their content and ratios are different. The former contains approximately twice the amount of Irganox 1010 than in pipe P490new, and a slightly higher content for the other two antioxidants. However, the pipe P061new contains only Irganox 1010 and Irgafos 168 in its composition, and the content of the last is twice that found in the other two pipes P826new and P490new.

Figure 9 also displays that the amount of all these antioxidants is considerably reduced (or absent) in the different aged pipes after their exposure to the aging treatment at 110 °C. This feature indicates that consumption of antioxidants has taken place during the test for evaluation of the long-term resistance to internal hydrostatic pressure at 110 °C. The loss has been complete in two of the pipes, the P490aged and P061aged ones, while the presence of Irganox 1010 and Irganox 1330 is still detected in the pipe P826aged, although in a low amount. These results are in agreement with those very low OIT values found for the P490aged and P061aged pipes, which are also very analogous (see in Figure 8), while those observed for either P826aged^IN^ or P826aged^OUT^ are sensibly higher because antioxidants are still present. Furthermore, these results could also explain the reason why the pipe P826aged is the only one that does not undergo failure at 110 °C in the time established by the standard ISO 1167.

It has to be said that the combination of antioxidants found for these PP-R pipes is in accordance with the ones used for the optimum protection of other polymeric materials, such as polyethylene, poly(acrylonitrile-butadiene-styrene) or styrene-butadiene copolymer [67,77,78].

This determination provides, when performed in either the new or the aged pipes, valuable information on the actual antioxidants present before and after the internal hydrostatic pressure tests at 110 °C. Nevertheless, this estimation does not allow distinguishing between the antioxidants located in the inner or outer sides of pipes, since evaluation (results represented in Figure 9) has been performed in the whole bulk of the pipes. Thus, it cannot be stated if the action of pressure, water and temperature in the inner part is more or less aggressive for the consumption of antioxidants than the effect of dry air and temperature in the outer counterpart. Furthermore, this assessment does not allow learning regarding the loss of antioxidants at their earlier stages: only at the beginning and at the end.

**Figure 10:**
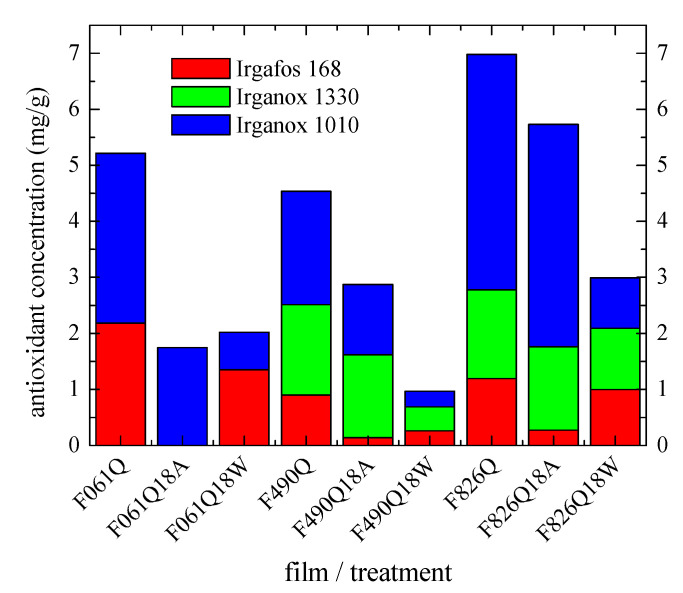
Influence of the aging environment media on the consumption of additives.

In order to overcome some of these drawbacks, a basic production replica was performed in an oven to determine the effect of temperature and environment (water or dry air) on the loss of antioxidants. Specimens treated at 110 °C under these two surroundings represent both sides of pipes. For that, some Q films prepared by compression molding from the corresponding new pipes applying a fast cooling were placed in an oven at 110 °C for 18 days (additional details in the Experimental section). Figure 10 shows the content of each antioxidant in the just processed Q films together with that in the ones directly exposed at 110 °C in air (labeled as Q18A) or in the films at 110 °C immersed in water (named as Q18W). Results are in a very good agreement for a given material when comparing the values obtained from the new pipe (see in Figure 9) and those derived from the Q film and shown in Figure 10. These results also agree with the ones achieved from the S films (not represented for clarity of reading), a fact indicating that the cooling rate applied during the film processing from the new pipes does not significantly affect the consumption rate of antioxidants.

Figure 10 also displays that the initial Q films exhibit, as expected, higher antioxidant contents than those found in the films aged at high temperature within the oven, independently of their exposure to warm dry air or hot water. Important differences are, however, observed regarding the consumption of each antioxidant for the films exposed to these two thermal environments.

As commented above, Irganox 1010 and Irganox 1330 are primary antioxidants. Irganox 1010 is the one found in the major amount in all the pristine Q films, similar to what was observed in the new pipes (see Figure 9). Its loss is significantly higher in all the films immersed in hot water in comparison with those just exposed to warm dry air. This result seems to indicate that Irganox 1010 consumption could be faster in the inner part if this behavior could be extrapolated to the one observed in the aged pipes. It has to be remembered that the antioxidant tests, whose results are represented in Figure 9, were performed as the average of inner and outer parts in the pipes.

A slight preferential consumption is also found for Irganox 1330 in the F826Q18W and F490Q18W films immersed in hot water. No conclusion can be obtained from the F061 film since it does not contain this additive. It is interesting to remark that Irganox 1330 loss is faster in the F490Q18W film than in the F826Q18W one, taking into account that its initial content in both films (F490Q and F826Q, respectively) is rather analogous. It seems that the higher amount existing in Irganox 1010 preserves the consumption of Irganox 1330 in the F826Q film when it is dipped in water.

An opposing trend is observed for the loss of the secondary Irgafos 168 antioxidant, i.e., its consumption is considerably more important in the films only exposed to warm dry air, independently of the PP-R pipe. In fact, it is completely consumed in the F061Q18A, even though its initial content in the F061Q film was sensibly higher. This feature could be related to absence of the Irganox 1330 antioxidant in the formulation of pristine P061new pipe. Irganox 1330 seems to have acted in a protective role for Irgafos 168 in this warm dry air environment, as deduced from the results obtained for F826Q18A and F490Q18A.

Irgafos 168 loss in the films immersed into hot water is, however, much slower. This fact points out its greater resistance in this specific atmosphere.

Irganox 1010 shows an oxidation capacity slightly higher than the Irganox 1330. This is in agreement with the literature [79] where hydrolysis is the preferred decomposition mechanism for Irganox 1010, while the Irganox 1330 consumption in water takes place through a gradual transformation into three different quinone structures. These species were also identified by our research team [15]. The stabilization ability of phenolic groups existing in the different oxidized species in Irganox 1330 has previously been demonstrated [80], so the reduction in content of parent species is not indicative of a drastic reduction in the antioxidant capacity of this additive.

**Figure 11:**
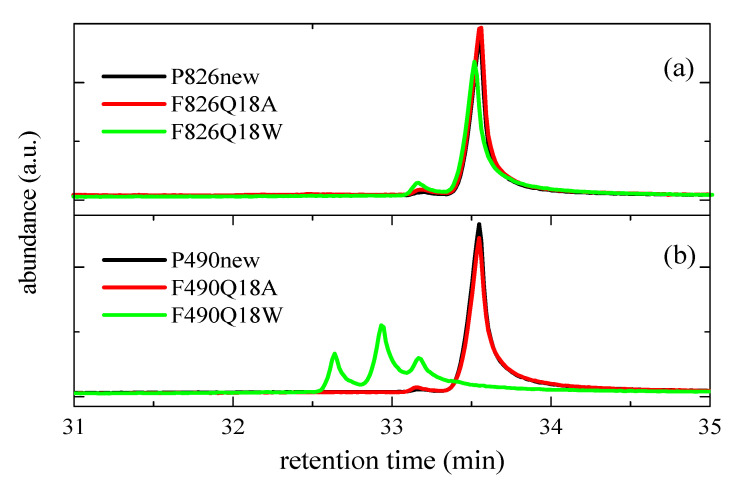
GC-MS chromatograms showing the degradation extent of Irganox 1330 in the indicated new pipes and aged films under two environments (warm dry air (Q18A) and hot water (Q18W)): (**a**) P826 and (**b**) P490.

Figure 11 depicts that the formation of these oxidized species (with retention times between around 32.6 and 33.2 min) is much more noticeable in the F490Q18W film immersed in water, also showing a significant reduction in the original form of Irganox 1330 compared with those corresponding to its oxidized compounds (where the predominant species involve two quinoid forms). In the case of pipe P826, that reduction is considerably smaller, although the formation of oxidized species is also slightly higher for sample F826Q18W than for F826Q18A.

It should be recalled at this point that consumption of antioxidants in the inner and the outer sides corresponds to different degradation processes, as seen from the OIT results represented in Figure 8, and further confirmed in Figure 10 by the tests performed from the films attempting to reproduce the two environments involved. Loss of Irgafos 168 is much faster in the film directly in contact with warm dry air than in that immersed in hot water, i.e., in the exterior is quicker than in the interior part, as revealed in Figure 10. In spite of the complete consumption of antioxidants in the pipe P061aged, as seen in Figure 9, the presence of the glass fiber layer could lead to a resistance to failure during the aging test to internal hydrostatic pressure longer than that exhibited by the pipe P490aged, where loss of antioxidants was also complete.

## 4. Conclusions

Distinct aspects were examined in several pipes to understand their different resistance to an internal hydrostatic pressure test performed at a high temperature for the prediction of their long-term failure behavior. The analysis was carried out in both the pipes aged in that pressure test and in the original new pipes. Firstly, all the pipes worked out analogously from either crystalline or phase transition standpoints. As an effect of the aging process undergone during the pressure test, a shift of T_m_^α^ to higher values and an increase in crystallinity were observed in all of the aged pipes.

Concerning the presence of fillers at each pipe and their identification, TiO_2_ was found in all of the pipes. In addition, the P490 pipe exhibited CaCO_3_ in its formulation, while a glass fiber layer was present in the bulk of the P061 pipe. These differences lead to variations in the morphological characteristics of the different pipes, turning out evidence of formation of voids in the P490 pipe, a fact that can contribute to enlarging its global heterogeneity.

OIT results were considerably different between the distinct new pipes. Performance of the pressure test on them also influenced the OIT response exhibited by the aged pipes. The P826 one, showing the highest OIT in the P826new pipe, also exhibited the largest values in P826aged after the aging test. The P490new pipe displayed the intermediate result and the lowest one was observed in the P061new. Nevertheless, changes that occurred during the aging internal hydrostatic pressure test provoked exhibition of a rather similar behavior for these two latest P490aged and P061aged pipes.

Determination of the loss of antioxidants by GC-MS was very conclusive. Irganox 1010, Irganox 1330 and Irgafos 168 were found in the P826new and P490new pipes, although in different contents and ratios. The P061new only incorporated Irganox 1010 and Irgafos 168 in its composition. The aging test for long-term failure estimation played a key role in the consumption of these antioxidants, in such a way that the loss was complete in the P490aged and P061aged pipes, while presence of Irganox 1010 and Irganox 1330 was still detected in the pipe P826aged, although in a low amount.

All of these results allow concluding the following: (A) The characteristic that led the P826 pipe to be the only one that overcame the test under a constant hydrostatic internal pressure of 6 bar at a temperature of 110 °C was found to be the greater amount of antioxidants added, which helped preserve its oxidation, together with the incorporation of only TiO_2_, which favored its ultimate homogeneity. (B) Presence of a glass fiber layer in the bulk of the P061 pipe was essential to almost allow it to bear the whole duration of the pressure test at high temperature. Although the P061 only contained two antioxidants in the new pipe and their consumption was complete during aging, the glass fiber layer partially hindered, or at least slowed down, the propagation of cracks developing during its more extensive chemical degradation associated with its complete antioxidant depletion. (C) The formation of holes observed in the P490 pipe, joined to an insufficient package in antioxidants (in spite of containing the three antioxidants present in the P826 pipe) provoked its premature failure, it being the pipe showing the shortest resistance time to the action of pressure at 6 bar and at 110 °C.

Even if the crystalline characteristics are appropriate in the polymeric matrix, the success of a pipe lies in the homogeneous dispersion of components for avoiding damage to interfacial properties, and in a correct cocktail of antioxidants used in its formulation.

## Figures and Tables

**Table 1:** Commercial labels and time before failure for the normalized aging test in the different PP-R pipes.

Sample	Time before Failure in the Aging Test (Hours)	Accomplishment of Standard ISO 1167
P061	8338	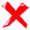
P490	6256	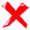
P826	8760	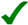

**Table 2:** Values of melting enthalpy (∆H_m_), crystallinity (f_c_^DSC^) and peak temperature (T_m_^α^), estimated from the first heating run, together with the crystallization temperature (T_c_) attained from the cooling process (both performed at a rate of 20 °C/min) for the inner and outer parts of either new or aged pipes.

Pipe	P061				P490				P826			
	∆*H*_m_ (J/g)	*f*_c_^DSC^	*T*_m_^α^ (°C)	*T*_c_ (°C)	∆*H*_m_ (J/g)	*f*_c_^DSC^	*T*_m_^α^ (°C)	T_c_ (°C)	∆*H*_m_ (J/g)	*f*_c_^DSC^	*T*_m_^α^ (°C)	*T*_c_ (°C)
new^IN^	87.3	0.55	142.0	109.0	96.4	0.60	143.0	109.5	90.3	0.56	142.5	107.5
new^OUT^	83.3	0.52	139.5	109.0	91.6	0.57	141.5	108.5	87.9	0.55	140.0	107.5
aged^IN^	100.9	0.63	146.0	109.0	99.0	0.62	148.5	110.0	102.0	0.64	146.5	106.5
aged^OUT^	101.8	0.64	143.5	108.5	93.9	0.59	144.0	109.5	96.6	0.60	143.0	107.0

## Data Availability

The data that support the findings of this study are available from the corresponding author upon reasonable request.

## Supplementary Materials

The following are available online at https://www.mdpi.com/article/10.3390/polym13162825/s1, Figure S1: Transversal cut of each individual new PP-R pipe; Figure S2: FTIR spectra of the obtained residues from the different pipes after their calcination; Figure S3: Thermo-oxidative behavior of different antioxidants under an oxidative atmosphere. The oxidation stage begins at 12.5 min; Table S1: Specimens analyzed in the present research and a brief description; Table S2: Data from SEM-EDX.
