# Supplementary material for: An Effective Package of Antioxidants for Avoiding Premature Failure in Polypropylene Random Copolymer Plastic Pipes under Hydrostatic Pressure and High Temperature
Source: Polymers (Basel). 2021 Aug 22;13(16):2825. doi: 10.3390/polym13162825 (PMC8400617; doi:10.3390/polym13162825)
Supplement: Supplementary file 1 [file polymers-13-02825-s001.zip › polymers-1275339-supplementary.pdf]

Supplementary Materials

An effective package of antioxidants for avoiding premature failure in polypropylene random copolymers plastic pipes under hydrostatic pressure and high temperature

Enrique Blázquez-Blázquez[1], Joaquín Lahoz[2], Ernesto Pérez[1], María L. Cerrada[1*]

[1]Instituto de Ciencia y Tecnología de Polímeros (ICTP-CSIC). Juan de la Cierva 3, 28006 Madrid, Spain.
[2]Centro de Ensayos, Innovación y Servicios, S.L. (CEIS). Cr. Villaviciosa de Odón a Móstoles (M-856) Km. 1.5, 28935 Móstoles, Spain.
E-mail address of corresponding author: mlcerrada@ictp.csic.es

Table S1. Specimens analyzed in the present research and a brief description.

Samples	Description
P061new	original pipe
P490new	original pipe
P826new	original pipe
P061aged^{IN}	interior part of pipe subjected to an accelerated aging test
P061aged^{OUT}	outer part of pipe subjected to an accelerated aging test
P490aged^{IN}	interior part of pipe subjected to an accelerated aging test
P490aged^{OUT}	outer part of pipe subjected to an accelerated aging test
P826aged^{IN}	interior part of pipe subjected to an accelerated aging test
P826aged^{OUT}	outer part of pipe subjected to an accelerated aging test
F061Q	rapidly cooled Q film
F061S	slowly cooled S film
F490Q	rapidly cooled Q film
F490S	slowly cooled S film
F826Q	rapidly cooled Q film
F826S	slowly cooled S film
F061Q18A	rapidly cooled Q film annealed at 110 °C as an attempt of reproducing the outer part (air) of an aged pipe
F061S18A	slowly cooled S film annealed at 110 °C as an attempt of reproducing the outer part (air) of an aged pipe
F490Q18A	rapidly cooled Q film annealed at 110 °C as an attempt of reproducing the outer part (air) of an aged pipe
F490S18A	slowly cooled S film annealed at 110 °C as an attempt of reproducing the outer part (air) of an aged pipe
F826Q18A	rapidly cooled Q film annealed at 110 °C as an attempt of reproducing the outer part (air) of an aged pipe
F826S18A	slowly cooled S film annealed at 110 °C as an attempt of reproducing the outer part (air) of an aged pipe
F061Q18W	rapidly cooled Q film immersed in water at 110 °C as an attempt of reproducing the inner part (water) of an aged pipe
F061S18W	slowly cooled S film immersed in water at 110 °C as an attempt of reproducing the inner part (water) of an aged pipe
F490Q18W	rapidly cooled Q film immersed in water at 110 °C as an attempt of reproducing the inner part (water) of an aged pipe
F490S18W	slowly cooled S film immersed in water at 110 °C as an attempt of reproducing the inner part (water) of an aged pipe
F826Q18W	rapidly cooled Q film immersed in water at 110 °C as an attempt of reproducing the inner part (water) of an aged pipe
F826S18W	slowly cooled S film immersed in water at 110 °C as an attempt of reproducing the inner part (water) of an aged pipe

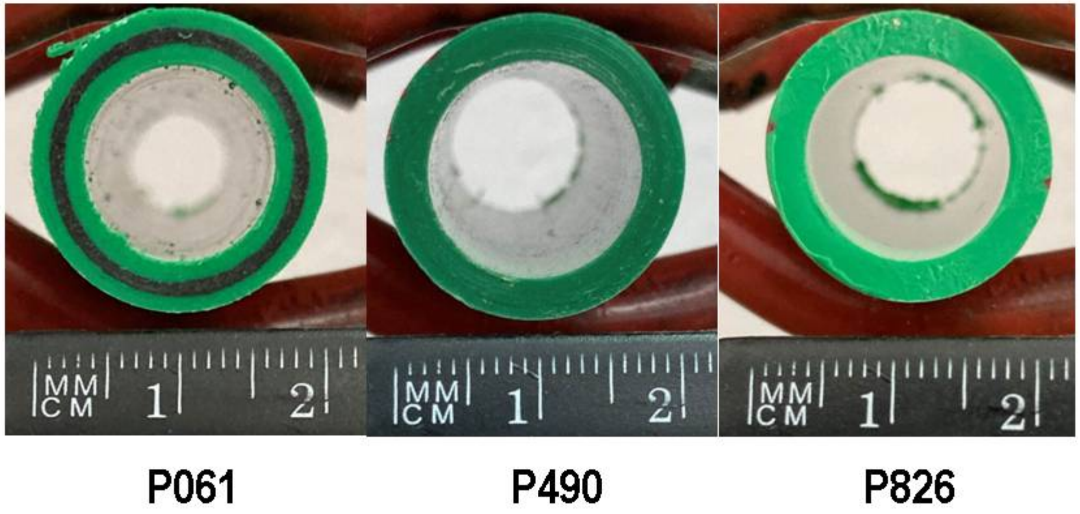

Figure S1. Transversal cut of each individual new PP-R pipe.

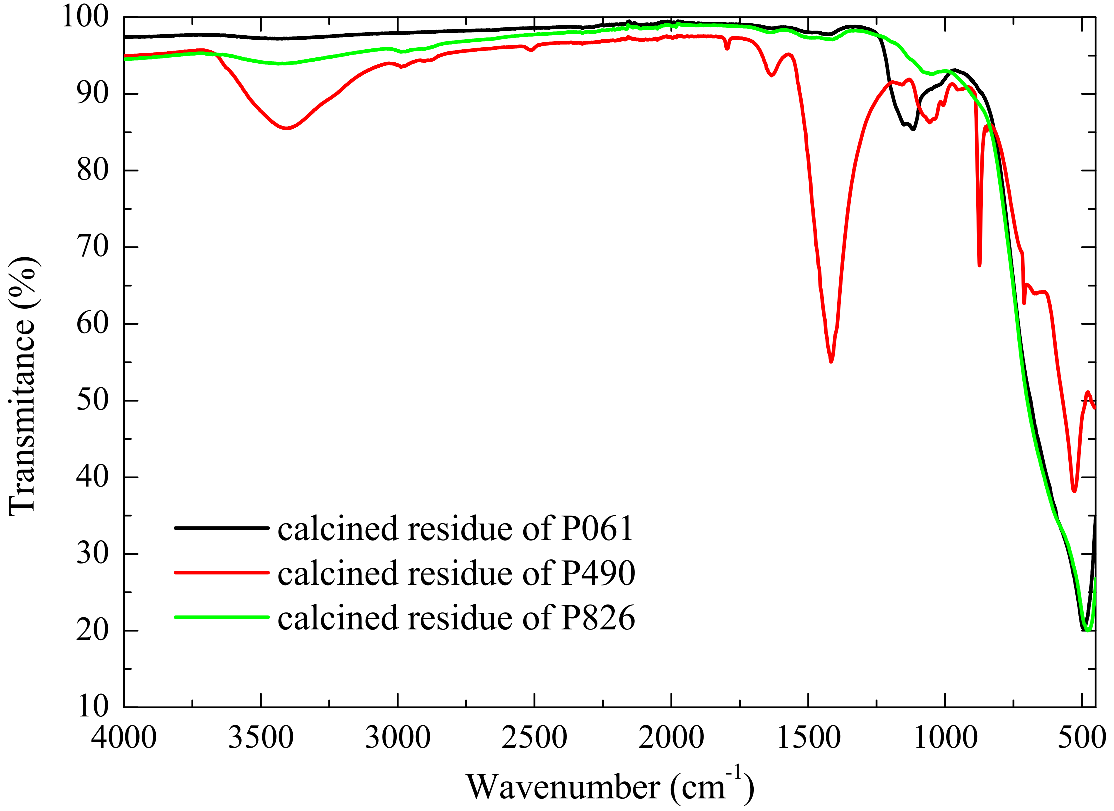

Figure S2. FTIR spectra of the obtained residues from the different pipes after their calcination.

Table S2. Data from SEM- EDX

Pipe	El	Series	norm. C (wt. %)	error (wt. %)
P061	C	K-series	0.28	0.1
	O	K-series	46.53	5.6
	Na	K-series	0.06	0.0
	Mg	K-series	0.05	0.0
	Al	K-series	1.07	0.1
	Si	K-series	22.72	0.0
	Cl	K-series	0.22	0.0
	Ti	K-series	29.07	1.8
P490	C	K-series	3.02	0.6
	O	K-series	34.56	4.6
	Na	K-series	0.00	0.0
	Mg	K-series	0.29	0.0
	Al	K-series	1.16	0.1
	Si	K-series	0.31	0.0
	P	K-series	0.40	0.0
	Cl	K-series	0.46	0.0
	Ca	K-series	20.84	0.7
	Ti	K-series	12.36	0.4
	Fe	K-series	18.20	0.6
	Zn	K-series	8.40	0.4
P826	C	K-series	1.71	0.6
	O	K-series	44.03	7.2
	Na	K-series	0.30	0.1
	Mg	K-series	0.13	0.0
	Al	K-series	1.22	0.1
	Si	K-series	0.31	0.0
	P	K-series	0.54	0.1
	S	K-series	0.17	0.0
	Cl	K-series	0.28	0.0
	Ca	K-series	1.11	0.1
	Ti	K-series	49.82	1.5
	Cu	K-series	0.38	0.0

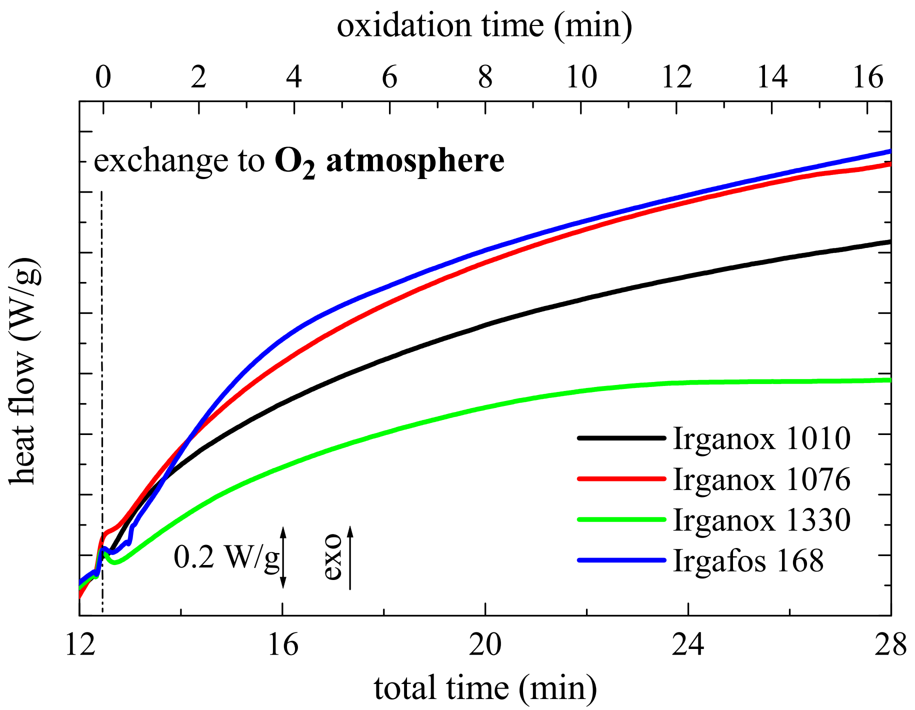

Figure S3. Thermo-oxidative behavior of different antioxidants under an oxidative atmosphere. The oxidation stage begins at 12.5 min.
